# The diagnostic value of multimodal imaging based on MR combined with ultrasound in benign and malignant breast diseases

**DOI:** 10.1007/s10238-024-01377-1

**Published:** 2024-05-23

**Authors:** Dong Bai, Nan Zhou, Xiaofei Liu, Yuanzi Liang, Xiaojun Lu, Jiajun Wang, Lei Liang, Zhiqun Wang

**Affiliations:** 1 Department of Radiology, Aerospace Center Hospital, Beijing, China; 2 Department of Ultrasound, Aerospace Center Hospital, Beijing, China; 3 Department of Radiology, Liangxiang Hospital, Fangshan District, Beijing, China

**Keywords:** Breast disease, Magnetic resonance imaging, Ultrasound, Radiomics, Multimodal

## Abstract

We aimed to construct and validate a multimodality MRI combined with ultrasound based on radiomics for the evaluation of benign and malignant breast diseases. The preoperative enhanced MRI and ultrasound images of 131 patients with breast diseases confirmed by pathology in Aerospace Center Hospital from January 2021 to August 2023 were retrospectively analyzed, including 73 benign diseases and 58 malignant diseases. Ultrasound and 3.0 T multiparameter MRI scans were performed in all patients. Then, all the data were divided into training set and validation set in a 7:3 ratio. Regions of interest were drawn layer by layer based on ultrasound and MR enhanced sequences to extract radiomics features. The optimal radiomic features were selected by the best feature screening method. Logistic Regression classifier was used to establish models according to the best features, including ultrasound model, MRI model, ultrasound combined with MRI model. The model efficacy was evaluated by the area under the curve (AUC) of the receiver operating characteristic, sensitivity, specificity, and accuracy. The F-test based on ANOVA was used to screen out 20 best ultrasonic features, 11 best MR Features, and 14 best features from the combined model. Among them, texture features accounted for the largest proportion, accounting for 79%.The ultrasound combined with MR Image fusion model based on logistic regression classifier had the best diagnostic performance. The AUC of the training group and the validation group were 0.92 and 091, the sensitivity was 0.80 and 0.67, the specificity was 0.90 and 0.94, and the accuracy was 0.84 and 0.79, respectively. It was better than the simple ultrasound model (AUC of validation set was 0.82) or the simple MR model (AUC of validation set was 0.85). Compared with the traditional ultrasound or magnetic resonance diagnosis of breast diseases, the multimodal model of MRI combined with ultrasound based on radiomics can more accurately predict the benign and malignant breast diseases, thus providing a better basis for clinical diagnosis and treatment.

## Introduction

The anatomy of the mammary gland consists of different blood vessels, connective tissue, milk ducts, lobules, and lymphatic vessels. When breast tissue grows abnormally and cell differentiation becomes uncontrolled, tumors form in the milk ducts or lobules. The developing tumor may be benign or malignant [1]. Breast cancer is the most common type of cancer and the biggest cause of death for women [2]. According to the statistics published by the World Health Organization (WHO), among 1,350,000 cases of breast cancer, there are 460,000 deaths worldwide every year [3]. The incidence of breast cancer has been on the rise for most of the last four decades, increasing by 0.5% per year in the most recent data year (2010–2019), driven primarily by localized breast cancer and hormone receptor-positive disease [4]. From 1975 to 1989, the overall breast cancer death rate increased by 0.4% per year, but by 2020 it had decreased by 43%. The decline in breast cancer mortality is attributed to better and more targeted treatment and early detection through screening breast imaging [5, 6].

With the development of imaging technology, a variety of imaging methods can be applied to the early screening of breast disease, such as breast X-ray mammography, magnetic resonance imaging, positron emission tomography, computed tomography, ultrasound.

Histopathology is the gold standard for the diagnosis of breast disease. Ultrasound has gradually become the main examination method of breast diseases because it can be diagnosed from different angles and directions in real time, without the radiation of breast diseases. MRI, especially enhanced MRI, can show the details of breast tissue in more detail, and make the diagnosis of suspicious lesions and the scope of invasion. However, there are many deficiencies in the diagnosis of breast cancer by conventional ultrasound or MRI alone. To a certain extent, there are overlapping images of benign and malignant breast tumors, and the lack of specificity of malignant tumors also leads to a decrease in the accuracy of clinical diagnosis of breast tumors, resulting in low early detection rate of breast cancer and loss of the best opportunity for treatment. This is also an important reason for the poor prognosis of some breast cancer patients. At the same time, manual examination of breast images may reduce diagnostic efficiency and increase misdiagnosis or missed diagnosis due to the large number of images or experience level. Therefore, it is very important to find new techniques and methods for the diagnosis of breast mass. In recent years, artificial intelligence technology has made great progress in the automatic analysis of abnormal detection of medical images. Compared with manual inspection, automatic image analysis based on artificial intelligence avoids tedious and time-consuming screening process and effectively captures valuable relevant information from a large amount of image data [7, 8].

Radiomics technology, which is developed from computer-aided detection/diagnosis (CAD) technology, can characterize the heterogeneity of tumors by mining massive quantitative imaging features to assist physicians in making clinical decisions [9]. In the processing and analysis of breast tumor images, radiomics methods have also been widely used. Generally speaking, the processing flow mainly includes image preprocessing, tumor segmentation, feature extraction and optimization, classification model construction and testing, and other steps [10]. With the continuous development of modern imaging technology, the comprehensive application of different examination methods is helpful to improve the accuracy of diagnosis, so as to provide better clinical services. Fusion multimodal imaging examination technology can provide more information support for the diagnosis of breast diseases, which is a new imaging examination scheme for the diagnosis of breast diseases. Multimodal examination combined with artificial intelligence is used to play the advantages of different examination techniques and improve the accuracy of clinical diagnosis. However, there are few studies on the multimodal examination methods of MR combined with ultrasound based on radiomics in benign and malignant breast diseases. ROC curve analysis was not carried out in the existing studies, and the diagnostic effectiveness of different diagnostic schemes could not be clearly presented. In view of this research status, this study will focus on breast diseases and explore the diagnostic efficacy of multimodal radiomics based on ultrasound alone, MRI alone, and combined ultrasound and MRI application, in order to provide more reference for the clinical diagnosis of breast diseases. In view of this research status, this study takes breast diseases as the main research object, and explores the diagnostic efficacy of radiomics based multimodal imaging examination methods of ultrasound alone, MRI alone, and combined ultrasound and MRI application, in order to provide more reference for the clinical diagnosis of breast diseases.

## Materials and methods

### General information

**Fig. 1:**
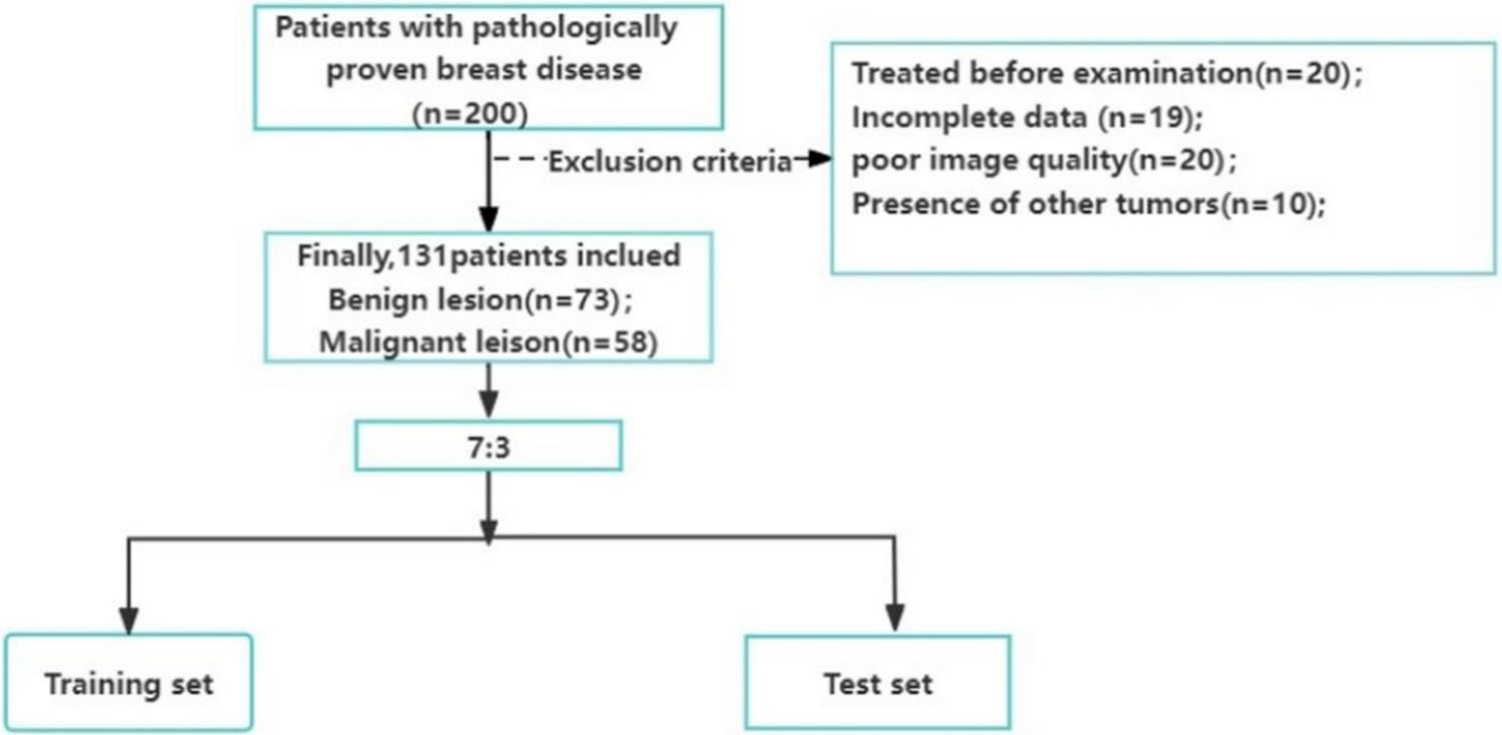
Patients selection flow chart

The clinical and imaging data of 200 patients with breast diseases confirmed by pathology in Aerospace Center Hospital from January 2021 to August 2023 were retrospectively analyzed, including 100 cases of benign diseases and 100 cases of malignant diseases. Inclusion criteria were: (1) benign and malignant breast diseases confirmed by surgery or needle biopsy; (2) enhanced breast MR and ultrasound before operation; (3) complete clinical data; (4) no history of other tumors.Exclusion criteria: (1) preoperative chemoradiotherapy or endocrine therapy; (2) The clinicopathological data is not perfect or the image quality is poor, and the artifacts are heavy; (3) There are other benign and malignant tumors; (4) Previous unilateral mastectomy. Finally, 131 cases were included in the study (Fig. 1). A total of 131 patients were randomly divided into training group (92 cases) and validation group (39 cases) according to the ratio of 7∶3. Clinical information was collected, including age and pathological type of breast disease. This study has been approved by the ethics committee of the Aerospace Center Hospital, and informed consent was waived because this study was a retrospective study and patient information was treated privately.

### MRI and ultrasound equipment parameters

All patients underwent a 3 T Siemens Skyra examination with a 16-channel breast specific phased-array surface coil, and the patient was placed in the prone position with the head entering the scanner first. The patient’s breast was naturally suspended in the coil, and the nipple was kept in the center of the coil.The scanning range was selected from both sides of the breast tissue, aorta and axilla.The specific scanning parameters were as follows: (1) axial T1WI parameters: Field of view (FOV) 360 mm × 360 mm, excitation frequency 3 times, matrix 320 × 320, interval of layers 1 mm, slice thickness 1.2 mm, flip angle 80°,TR 5.37 ms, TE 2.46 ms; axial STIR T2WI parameters: TE 63 ms, TR 3,700 ms, Slice thickness 4 mm; (2) diffusion weighted imaging (DWI) was performed with a single excitation spin-echo echo planar imaging (SE-EPI) with the following parameters: FOV 360 mm × 360 mm, slice thickness 5 mm,TE 58 ms, excitation times 6 times, interval 1 mm,TR 5980 ms,b values selected as 0 and 1 000 s/mm^2^, and apparent diffusion coefficient (ADC) was calculated. (3) Dynamic enhanced MRI adopted the 3D rapid gradient echo sequence, The parameters were as follows: NEX 0.7, FOV 350 mm × 350 mm, No-interval scan, Slice thickness 1 mm, TR 4.36 ms,TE 1.56 ms. After the normal scan was completed, dynamic enhancement scan was performed. A high pressure syringe was used to inject 0.2 mmol/kg of contrast agent gadolinium pentic acid into the intravenous mass at the rate of 2 mL/s, and 20 mL of normal saline was injected at the same rate. Then dynamic scanning was performed in 7 scanning phases, the first phase was the enhanced front mask, and the second phase was the enhanced phase. The acquisition time of each dynamic phase was 60 s.

All patients underwent breast ultrasound examination within 1 week before surgery. LOGIQ precise E9 ultrasonic diagnostic instrument with probe frequency of 6 ~ 15 MHz was used. The patient was placed in the horizontal position, the probe was placed vertically on the breast skin surface, and the probe was scanned radially with the nipple as a multi-section plane. On the basis of obtaining a good two-dimensional image, the maximum surface of the nodules was displayed by superimposing color and energy Doppler blood flow imaging. According to the size and shape of the nodules, the maximum long-axis section of the lesion was stored in the image in the form of DICOM. All images were collected and evaluated by two physicians with more than 6 years of experience in breast ultrasound diagnosis, and the final classification results were obtained. The frequency, focus, gain and time compensation curve were adjusted to achieve the best image quality.

### Image acquisition and radiomics feature extraction

**Fig. 2:**
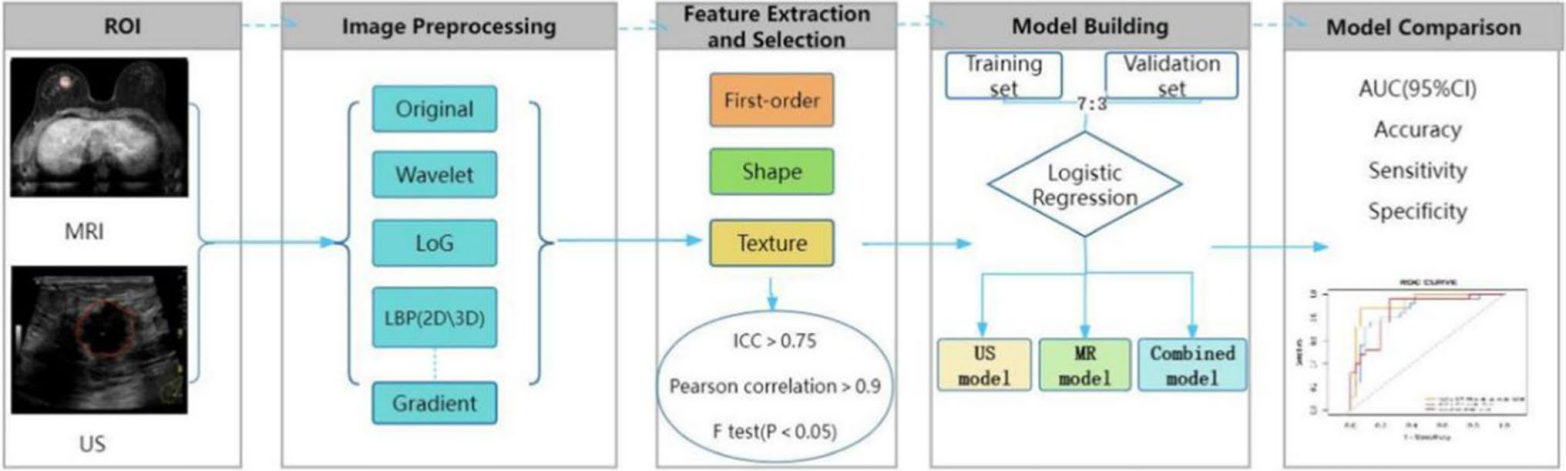
The workflow of the radiomics process

Ultrasound and MRI enhanced early images were imported from the picture archiving and communication system (PACS) system as “*.DICOM” into the Deepwise Multimodal Research Platform version 2.2 (https:// keyan.deepwise.com) (Beijing Deepwise and League of PHD Technology Co., Ltd, Beijing, China). It is an integrated machine learning platform for medical data analysis using the mature python pyradiomics (version 3.0.1) and scikit-learn (version 0.22) packages. A radiologist and sonographer (Reader A, with 6 years of experience in breast disease diagnosis and no knowledge of pathological findings) reviewed all images and semi-automatically mapped the area of interest (ROI), a senior radiologist and sonographer (Reader B, with 16 years of experience in breast disease interpretation, Also unaware of the pathology) reviewed all ROI for viewer A segmentation. Viewers A and B negotiated the controversial case to reach an agreement. Fifty lesions were randomly selected and resegmented by the senior radiologist and sonographer (reader B) to calculate the interclass correlation coefficient (ICC). Features with an ICC > 0.75 were considered to have good agreement. The specific flow chart of radiomics analysis in this study was as follows (Fig. 2).

To eliminate the scale differences in image radiomics features, feature standardization was performed before feature selection. For feature selection, ten preprocessed image methods were selected including the raw filtering, laplacian of gaussian (LoG) filtering, wavelet filtering, gradient calculation, local binary patterns (LBP) in both 2D and 3D, square, square root, logarithm, and exponential. The features analyzed in this study included first-order features, shape-based features and texture features, including gray level co-occurrence matrix (GLCM), gray level run-length matrix (GLRLM), gray level size zone matrix (GLSZM), neighborhood gray-tone difference matrix (NGTDM), gray level dependence matrix (GLDM). We conducted feature correlation analysis and eliminated features with correlation coefficient below 0.9.When the linear correlation coefficient between any two independent variables on the training set was greater than this threshold, one of the features would be removed, which aimed to reduce the redundancy between features. The dependent variable had high priority retention rate and high linear correlation coefficient. The features through feature correlation analysis were used for further model training. After the feature correlation analysis, we chose F Test as the method of feature selection, that was, we divided features into different populations according to different label categories, and tested whether there were significant differences in the mean values of features among different populations. Features with significant differences were considered to be distinguishable for classification and would be retained.

### Model building

Following feature selection, the radiomics model was built using the Logistic Regression machine learning algorithm. After feature selection, a radiomic model was built using a Logistic Regression machine learning algorithm. Logistic regression is a typical model that uses regression methods to obtain binary classification results. All datasets were randomly split and divided into training set and validation set with the ratio of 7:3. Machine learning models for benign and malignant breast diseases recognition were constructed, including ultrasound model, MRI model, and ultrasound combined MRI model. The performance of the model was evaluated by area under the curve (AUC) of receiver operating characteristic (ROC), sensitivity, specificity, and accuracy.

### Statistical analysis

Statistical analysis was performed using SPSS version 22.0 (SPSS Inc. Chicago, IL).Quantitative variables were reported as mean ± standard deviation or interquartile median, while categorical variables were described as counts and percentages. Normal distribution was checked using the Kolmogorov–Smirnov test. To compare radiological features between benign and malignant breast lesions, quantitative variables used the Student *t* test or Mann–Whitney U test, and the Chi-square test for categorical variables. AUC, accuracy, sensitivity, and specificity were used to describe the classification performance of the different models. The intra-class correlation coefficient (ICC) for the intra-and inter-observer agreement was calculated. *P*-value < 0.05 was considered statistically significant.

## Results

### Clinical results

In this study, 200 patients were retrospectively analyzed, 69 of whom were excluded due to poor image quality (*n* = 20), lack of complete clinical or pathological data (*n* = 19), preoperative treatment (*n* = 20), and combination of tumors at other sites (*n* = 10). Finally, 131 patients with breast disease were included in the study. The patients ranged in age from 26 to 76 years old, with an average age of 49.11 ± 9.85 years. There were 73 cases of benign breast lesions, including 45 cases of fibroadenoma, 20 cases of adenopathy and 8 cases of intraductal papilloma. There were 58 malignant lesions, including invasive ductal carcinoma in 26 cases, ductal carcinoma in situ in 27 cases, and papillary carcinoma in 5 cases (Table 1).

**Table 1:** General clinical and pathological data of breast patients

Clinical factors	Benign cases (*n* = 73)	Malignant cases (*n* = 58)
Age (years)	40.02 ± 11.54	58.20 ± 8.16
Pathological results (*n* = 131)	Fibroadenoma (*n* = 45) Adenopathy (*n* = 20) Intraductal papilloma (*n* = 8)	Invasiveductalcarcinoma (*n* = 26) Ductal carcinoma in situ (*n* = 27) Papillary carcinoma (*n* = 5)

### Radiomics feature analysis

**Fig. 3:**
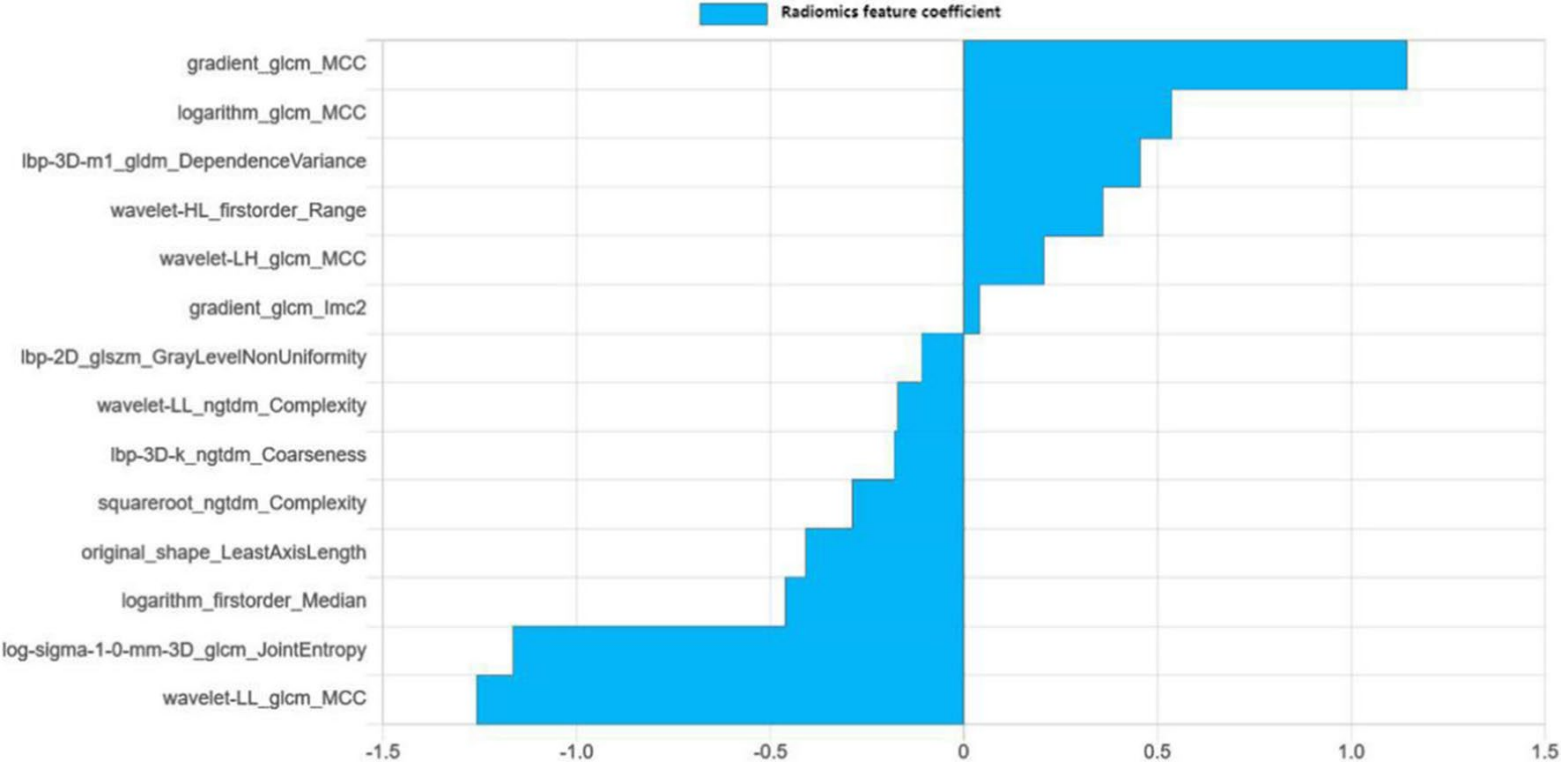
14 radiomics features coefficient in the combined model

The logistic regression classifier was used to establish three models of ultrasound, MR and ultrasound combined with MR. The inter-class and intra-class correlation coefficients of the features extracted by ultrasound were analyzed, and 1316 robust features were obtained. The F test was used to screen out 20 optimal features, including 1 first-order feature, 4 shape features and 15 texture features. For MRI, after ICC analysis, 1781 features with consistency greater than 0.75 were retained. After feature correlation analysis, 11 best features were selected by F-test, including 5 first-order features, 1 shape features and 5 textural features. In order to model the fusion of ultrasound and enhanced MRI, the features of the two sequences were combined to obtain a total of 3097 joint features. Then, 14 features with the most diagnostic value were selected by F test. In terms of feature types, it includes 2 first-order features, 1 shape feature and 11 texture features. The weight diagram of the features used for modeling is shown in Table 2 and Fig. 3.

**Table 2:** 14 radiomics features and their weights in the combined model

Radiomic features	Coef	Relative weight
gradient_glcm_MCC	1.1439	0.9092
logarithm_glcm_MCC	0.5373	0.4270
lbp-3D-m1_gldm_DependenceVariance	0.4559	0.3624
wavelet-HL_firstorder_Range	0.3598	0.2860
wavelet-LH_glcm_MCC	0.2063	0.1639
gradient_glcm_Imc2	0.0408	0.0324
lbp-2D_glszm_GrayLevelNonUniformity	− 0.1090	− 0.0866
wavelet-LL_ngtdm_Complexity	− 0.1716	− 0.1364
lbp-3D-k_ngtdm_Coarseness	− 0.1798	− 0.1429
squareroot_ngtdm_Complexity	− 0.2878	− 0.2288
original_shape_LeastAxisLength	− 0.4083	− 0.3245
logarithm_firstorder_Median	− 0.4607	− 0.3661
log-sigma-1–0-mm-3D_glcm_JointEntropy	− 1.1637	− 0.9248
wavelet-LL_glcm_MCC	− 1.2582	− 1.0000

### Comparison of three machine learning models

**Fig. 4:**
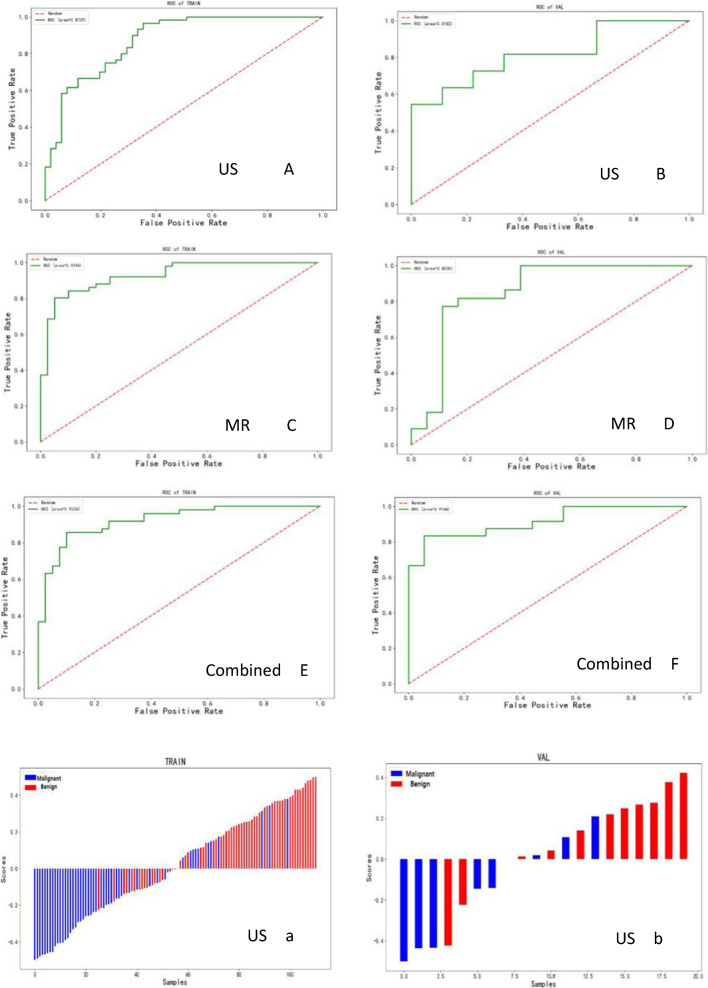

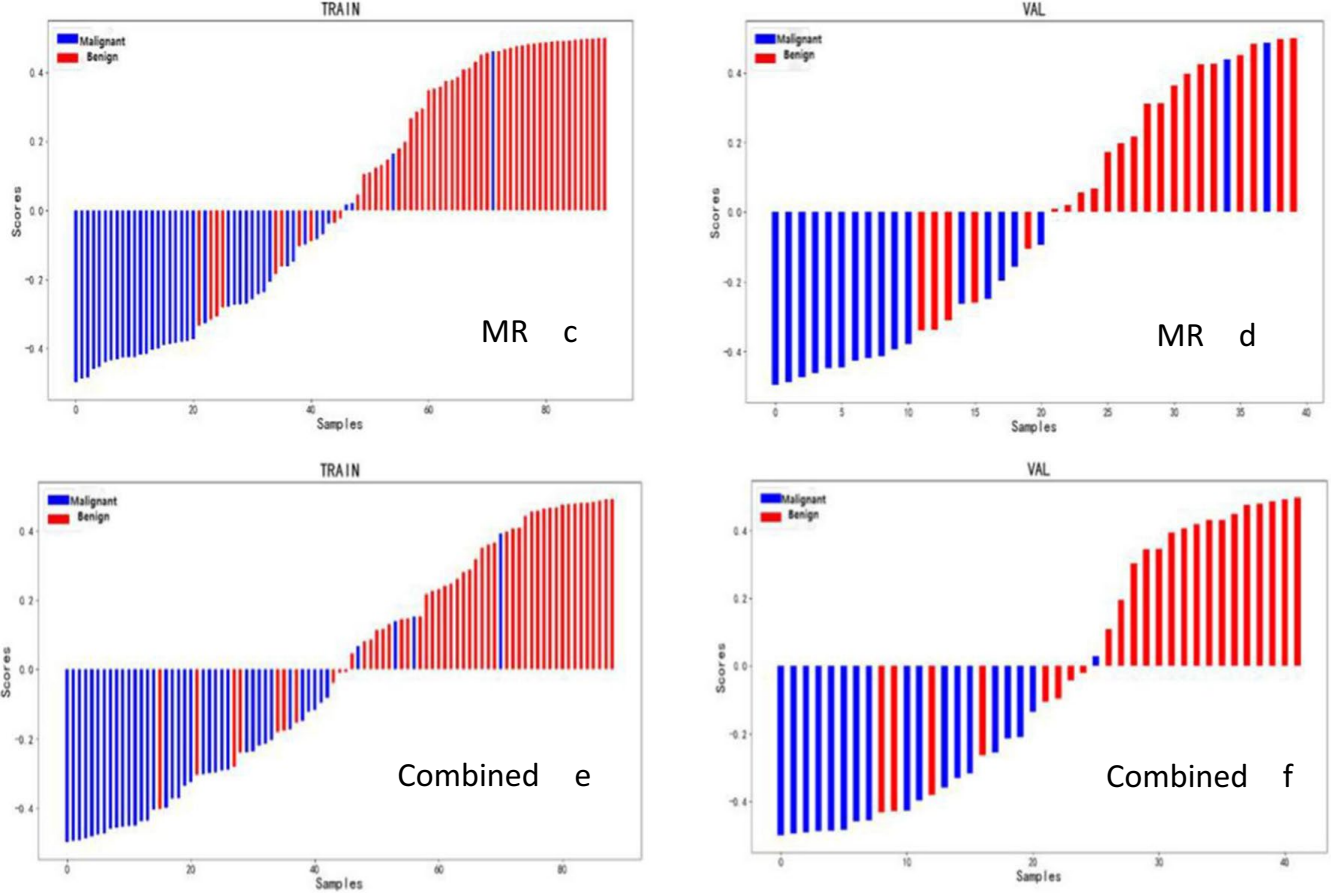
The ROC curves (**A**–**F**) and the waterfall plots (**a**–**f**) of the ultrasound model, MR model and combined model in the training set and validation set

**Fig. 5:**
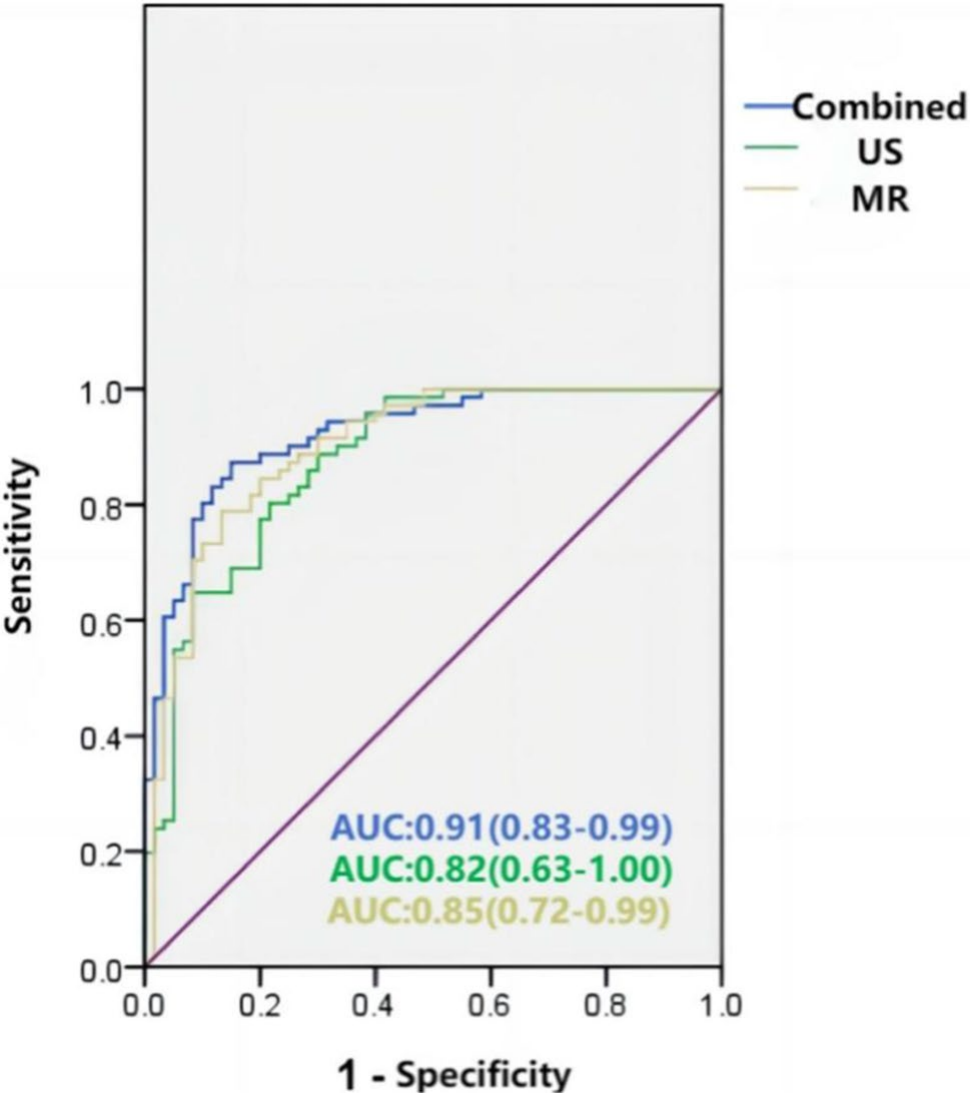
ROC curves of logistic regression model of different data modalities (US model; MR model and combined model)

The AUC values, accuracy, sensitivity, and specificity values of the ultrasound model, MR model, and ultrasound combined MR model are summarized in Table 3, and the ROC curves and waterfall plots of the training and validation sets of each model are shown in Fig. 4. In summary, the ultrasound combined with MR image combined model based on the Logistic Regression classifier has the best diagnostic efficiency, with AUC of 0.92 and 0.91, sensitivity of 0.80 and 0.67, specificity of 0.90 and 0.94, accuracy of 0.84 and 0.79, respectively, in the training group and the validation group. It was superior to either the ultrasound model alone or the MR Model alone (Fig. 5).

**Table 3:** Comparison of diagnostic efficiency of different models for breast diseases

	US model		MR model		Combined model	
	Training set	Validation set	Training set	Validation set	Training set	Validation set
AUC (95%CI)	0.87 (0.81–0.94)	0.82 (0.63–1.00)	0.93 (0.88–0.98)	0.85 (0.72–0.99)	0.92 (0.87–0.98)	0.91 (0.83–0.99)
Accuracy	0.76	0.70	0.85	0.83	0.84	0.79
Sensitivity	0.73	0.82	0.80	0.77	0.80	0.67
Specificity	0.78	0.56	0.90	0.89	0.90	0.94

## Discussion

Breast cancer is the most common cancer among women in 154 countries and the leading cause of cancer-related death in 103 countries [11]. According to the American Cancer Society, the average lifetime risk of developing breast cancer in a U.S. woman is estimated to be 12.3% (or 1 in 8 women), and by 2023, 300,590 invasive breast cancers (299,540 in women and 2,800 in men) and 55,720 cancers in situ in women will be diagnosed in the U.S.A About 43,700 people will die from breast cancer in 2023 [12]. Breast cancer is a serious public health problem. Early detection and control can reduce the death rate of this deadly disease. At present, the diagnosis and screening of breast diseases are mainly through mammography, ultrasound and magnetic resonance examination, and different examination methods have their own advantages and disadvantages. At the same time, because the analysis of image data involves the visual perception and cognitive ability of radiologists, the diagnosis of breast imaging-reporting and data system (BI-RADS) 3 score and 4a score appeared. Therefore, in order to make a definitive diagnosis, patients often undergo painful and expensive biopsy procedures. In this case, the artificial intelligence from single or multiple medical imaging model of multiple quantitative traits, highlight the image characteristics of invisible to the naked eye, significantly enhance the distinction between medical imaging and predict potential. In recent years, there have been many studies on non-invasive discrimination of benign and malignant breast lesions using artificial intelligence technology, including different machine learning, deep learning and data mining algorithms related to breast cancer prediction, detection of gene mutations in breast cancer, and application of deep learning to breast cancer with different imaging modalities. Their discrimination ability and work efficiency are usually better than that of radiologists [13–15].

KIM et al. [16] conducted an international study based on mammography, and the results showed that artificial intelligence models were superior to radiologists in tumor detection, asymmetric and distorted structure recognition. Using artificial intelligence technology, Hou Yin et al. [17] built a machine learning model of breast ultrasound images, and the diagnostic accuracy was better than that of imaging doctors. Artificial intelligence plays an important role in improving the diagnostic performance of breast MRI. The artificial intelligence breast classification system was applied to multi-parameter MRI examination, and the maximum AUC reached 0.852, and the diagnostic specificity was improved [18]. The study of Deng Qiuxiang et al. showed that ultrasound combined with mammography significantly improved its sensitivity (74.07%), specificity (88.61%) and accuracy (84.9%) [19]. At present, there are few studies on the application of multi-modal imaging methods based on radiomics in the diagnosis of breast lesions. Multi-modal imaging technology adopts more than two methods to make up for the shortcomings of a single imaging mode and improve diagnostic efficiency, which has become an important direction of the development of imaging diagnosis.

In this study, early enhanced MRI and ultrasound images were selected for feature extraction. MRI has great value in breast cancer diagnosis because of its high resolution of soft tissue and multi-sequence imaging. In view of the fact that dynamic contrast enhanced MRI (DCE-MRI) can reflect functional information such as hemodynamics of cancer foci, most imaging omics studies are carried out based on this [20, 21]. In the process of selecting the best features, there are six screening methods, including F test, Pearson, mutual information, L1 based, tree based, recursive, finally, the system selects the best feature screening method—feature screening F test (ANOVA F-value) based on analysis of variance, that is, features are divided into different populations according to different label categories, and the mean values of features between different populations are tested to see whether there are significant differences. Features with significant differences considered to be distinguishable for classification will be retained. Finally, 1316 robust features were obtained from the ultrasound model, and 20 optimal features were selected by F test, among which texture features accounted for 70%. The MRI model retained 1781 features. After feature correlation analysis, F-test was used to select 11 best features, among which texture features accounted for 45%.A total of 3097 joint features were obtained by the ultrasound combined with MR model. Then, 14 features with the most diagnostic value were selected by F test, and most of them were the features screened in the MRI model. Texture features accounted for 73% of the feature types. Texture features accounted for the majority of the three models, but texture features are rarely observed by the naked eye, suggesting that texture features have important clinical significance in the differential diagnosis of benign and malignant breast lesions. Texture features represent the pixel arrangement and spatial pixel relationship of the lesion image, and represent the texture heterogeneity of the growth and development of the lesion [22]. Texture features mainly include gray level co-occurrence matrix (GLCM), gray level size zone matrix (GLSZM), gray level run length matrix (GLRLM), neighbouring gray tone difference matrix (NGTDM) and gray level dependence matrix (GLDM). GLCM describes texture by calculating the condition of two pixels maintaining a certain distance on the image with a certain gray level, respectively, that is, the spatial correlation characteristics of gray level. GLSZM records the number of occurrences of the same adjacent elements in a two-dimensional region on the image. GLRLM describes the texture by recording the occurrence of multiple identical pixel values in the direction of the image, namely the grayscale path matrix. GLDM calculates the situation where the difference between a certain pixel and surrounding pixels on the image is less than a certain threshold, that is, the gray dependence matrix, to describe the texture. NGTDM calculates the difference between the average gray value of each pixel on the image and all pixels in its neighborhood, that is, the neighborhood gray difference matrix, to calculate the texture. In this study, GLCM is the most important texture feature. PARK et al. [23] studied radiomics features as a predictor of distant disease free survival in patients with invasive breast cancer. It was found that texture features based on GLCM/ gray-level size zone matrix (GLSZM) could capture heterogeneous texture information better than those based on histogram. The reason is that the texture feature based on GLCM/GLSZMs takes into account the interaction between adjacent pixels, which is very suitable for quantifying tumor texture and heterogeneity. The results of this study are consistent with those reported in the literature. In addition, the importance of wavelet features is also high, which indicates that the overall gray level of the image has a certain relationship with the differentiation of benign and malignant breast lesions. Some studies have reported that wavelet and Gaussian filters decompose the original image from different directions, which further presents multi-dimensional spatial heterogeneity and helps to reveal the tumor heterogeneity not detected in the original image [24, 25]. Wavelet features can reflect more information about tumor heterogeneity [26–29]. However, whether texture features and wavelet features can be used as specific imaging biomarkers to predict breast disease heterogeneity still needs to be verified by further multi-center and large-sample clinical trials.

In this study, ultrasound model, MR Model, and joint model were established based on Logistic Regression classifier. Logistic regression is a classification model, which is represented by conditional probability distribution in the form of parameterized logistic distribution. Here, the random variable X takes the value of a real number, and the random variable Y takes the value of 1 or 0. We estimate the model parameters by supervised learning method. Yassin et al. [30] outlined different traditional machine learning algorithms for breast cancer diagnosis in recent years in their review. These algorithms include decision trees (DT), random forests (RF), support vector machines (SVM), naive Bayes (NB), K-nearest neighbor (KNN), linear discriminant analysis (LDA), and logistic regression (LR). Some studies have shown that the Logistic Regression classifier has better diagnostic performance, and this study is consistent with literature reports. In this study, the training set of these three radiomics models showed high diagnostic efficiency, with the minimum AUC of 0.87 and the maximum AUC of 0.93. The AUC values in the validation set were lower than those in the training set, indicating that the model has good generalization ability and can ensure that the training group model still has good performance on the validation group. The logistic regression classifier-based ultrasound combined with MR model showed the best diagnostic performance, with an AUC of 0.91 in the validation group, which was better than the single ultrasound model (AUC of 0.82) or the single MR model (AUC of 0.85). These results indicate that the diagnostic efficiency of multimodal imaging based on radiomics is better than that of single imaging mode in the diagnosis of breast diseases. Ultrasound and X-ray mammography are the preferred imaging methods for early screening and diagnosis of breast diseases. With the advantages of real-time imaging, non-invasive, rapid and simple, high cost-effectiveness, high patient compliance, and high accessibility, breast ultrasound has become the most commonly used tool for breast tumor screening and diagnosis, and can detect changes in organ tissues and blood flow. However, due to the low resolution of ultrasonic instruments, it is impossible to accurately distinguish small lesions, and two-dimensional gray scale ultrasound cannot display tissue attenuation changes, which is easy to miss diagnosis and misdiagnosis.

MRI uses low-energy radio waves and intense magnetic fields to obtain detailed images of the internal structure of the breast, which can detect malignant breast lesions that cannot be detected by clinical, mammography and ultrasound. MRI has high accuracy in the diagnosis of breast diseases, and MRI features account for a large proportion of the 11 texture features of the fusion model with this study. However, breast MRI still has certain limitations, which is not sensitive to small calcification. The requirement of magnetic field uniformity is high; the examination is time-consuming and expensive. MRI examination has high sensitivity and low specificity, which is easy to cause false positive diagnosis and overestimate the scope of the lesion. Therefore, the combination of MR and ultrasound is more conducive to the diagnosis of breast diseases, and the integration of radiomics further improves the accuracy of lesion diagnosis. At present, there are few multi-modal radiomics studies. Zhang et al. [31] used T1WI, T2WI, DWI and DCE-MRI to establish a radiomics nomogram based on multi-parametric MRI to predict the lymphatic vascular invasion and clinical prognosis status of patients with breast invasive ductal carcinoma. Mu Xiaojing et al. found that the AUC of ultrasound in the diagnosis of benign and malignant BI-RADS 4 nodules was 0.923 (95%CI 0.904–0.942), and the AUC of mammography in the diagnosis of benign and malignant BI-RADS 4 nodules was 0.904 (95%CI 0.883–0.926).The AUC of ultrasound combined with mammography in the diagnosis of breast BI-RADS 4 tumors was 0.950 (95%CI 0.936–0.965), which was higher than that of single diagnosis [32]. Sui Guangping et al. [33] conducted a study on 70 patients with early breast cancer. The patients underwent combined examination of conventional ultrasound (as the routine group) and light scattering imaging + elastic imaging + contra-ultrasound + molybdenum target + magnetic resonance imaging (as the combined group), and compared with postoperative pathological results, respectively. It is found that the combined application of multimodal imaging technology can significantly improve the diagnostic accuracy of early breast diseases, which is worthy of promotion and application. However, there are few studies on the diagnostic value of ultrasound combined with MR Model based on radiomics for breast diseases. The combined application of several methods can play the role of complementarity and complementarity, which is of great significance to improve the accuracy of diagnosis, which is the innovation of this study.

There are still some shortcomings in this study: First, it is a retrospective study with a small sample size and unbalanced distribution of patient data, which may lead to bias in the study results; second, only T1W enhanced images were selected for MRI images, and other sequence images were not included, which may not fully exploit the advantages of mpMRI; finally, this study is a single-center study, and multi-center big data research is needed to improve the accuracy of the results in the future.

In summary, with the deep integration of artificial intelligence and medical technology, machine learning has reflected an important value in the processing and analysis of image depth. At the same time, combining the advantages of various imaging methods, fully exploiting the potential of AI-based multimodal imaging methods can further improve the value of imaging in the diagnosis of breast lesions.

## Data Availability

The datasets used and analyzed during the current study are available from the corresponding author on reasonable request.
